# Comparison of internal fit of metal‐ceramic crowns in CAD/CAM and lost‐wax techniques in all fabrication stages through replica weighting, triple scanning, and scanning electron microscope

**DOI:** 10.1002/cre2.529

**Published:** 2022-02-20

**Authors:** Sina Mohammadi Sadr, Elham Ahmadi, Masoumeh Hasani Tabatabaei, Saba Mohammadi, Faezeh Atri

**Affiliations:** ^1^ Department of Operative Dentistry, School of Dentistry Tehran University of Medical Sciences Tehran Iran; ^2^ Dental Research Center, Dentistry Research Institute Tehran University of Medical Sciences Tehran Iran; ^3^ School of Dentistry Tehran University of Medical Sciences Tehran Iran; ^4^ Department of Prosthodontics, School of Dentistry Tehran University of Medical Sciences Tehran Iran

**Keywords:** CAD/CAM, internal fit, lost wax, metal ceramic alloys

## Abstract

**Objectives:**

Restoration fit is one of the prerequisites of clinical durability. It is controversial as to whether computer‐assisted design/computer‐aided milling (CAD/CAM) or lost‐wax fabrication methods result in more fit metal‐ceramic crowns. This in‐vitro study was conducted to examine the internal fit of porcelain fused to metal crowns fabricated using CAD/CAM and lost‐wax techniques during fabrication stages (framework, porcelain, cementation) through digital triple scanning, replica weighting, and observation with electron microscopy.

**Material and methods:**

Twenty uniform resin dies of prepared first maxillary molars were randomly divided into two groups according to the fabrication technique: lost wax and CAD/CAM. The internal fit was measured in all steps of completing the crowns (framework, porcelain, and cementation) using different methods, including triple scanning, replica weighting, and scanning electron microscopy. The data were statistically analyzed using *t* test, Pearson, and repeated measures analysis of variance (*α* = .05).

**Results:**

Triple scanning revealed no difference in the internal fit of CAD/CAM and lost‐wax groups in all the fabrication steps (*p* > .05). The replica weighting method showed no difference between groups in the framework step (*p* > .05), while the internal fit was significantly better in the CAD/CAM group after porcelain application (*p* < .05). After cementation, electron microscopy measurements showed no difference between CAD/CAM and lost wax groups (*p* > .05). The Pearson correlation test showed no significant correlation between electron microscopy, replica weighing, and triple scanning methods (*p* > .05).

**Conclusion:**

According to scanning electron microscopy as the superior evaluation method, the internal fit of cobalt–chrome PFM crown of both CAD/CAM and lost wax groups was within the acceptable clinical range and there was no significant difference between them. Triple scanning revealed no difference in the internal fit of framework and porcelain steps but a better fit after cementation. According to replica weighting, the internal fit in the porcelain step was higher than the framework.

## 1 INTRODUCTION

Metal‐ceramic crowns (porcelain fused to metal: PFM) are widely used in fixed dental prostheses because of their mechanical properties (Jung, 2017; Kaleli & Saraç, 2017). The clinical durability of these restorations depends on the fit of the restoration to the abutment tooth (Jung, 2017). In case of improper fit, dissolution of the cement may occur, resulting in sequels, such as decay (Jung, 2017), periodontal disease (Kaleli & Saraç, 2017; Kane et al., 2015; Nawafleh et al., 2013; Nesse et al., 2015), inflammation of the dental pulp (Nawafleh et al., 2013), decreased long‐term success (Nawafleh et al., 2013), and loss of PFM crowns (Kane et al., 2015).

Conventionally, the framework of PFMs was mostly made of gold alloys, silver‐palladium, and nickel‐chromium. ADA Science & Research Institute (2021) Concerns about the toxicity of nickel and beryllium and the costs of gold and silver have resulted in the use of cobalt–chromium alloys as a replacement. Kane et al. (2015) reported that cobalt–chromium alloy is widely used because of its relatively low price (Kim et al., 2017; Nesse et al., 2015), stability in biological environments, corrosion resistance (Kane et al., 2015; Kim et al., 2017; Nesse et al., 2015; Viennot et al., 2005), and ease of fabrication using advanced digital methods (Nesse et al., 2015).

A metal framework is commonly fabricated using lost wax and casting, which is associated with a considerable number of laboratory steps (Kaleli & Saraç, 2017; Nesse et al., 2015), limited abilities to standardize the cement thickness (Nesse et al., 2015) and increased probability of laboratory errors. Today, to overcome the lost‐wax problems, digital methods and computer‐assisted design/computer‐aided milling (CAD/CAM) systems are increasingly used, which have the advantages of eliminating excessive human work, simplified implementation (Jung, 2017), and cost‐effectiveness (Kim et al., 2017). However, high equipment costs, equipment wear, and being time‐consuming are some of their limitations (Nesse et al., 2015).

Early CAD/CAM systems produced restorations with the improper internal fit (Kokubo et al., 2006; Peñate et al., 2015). However, these problems were minimized with the advent of technology in new systems. The fit of a prosthesis made by the CAD/CAM system depends on the quality and accuracy of the system set (Karaokutan et al., 2015). Internal fit is one of the important criteria for the clinical success of a restoration (Terry, 2002).

Internal fit is the degree of discrepancy between the internal surfaces of the restoration and the external surfaces of the tooth (Bicer & Unver, 2018). The regular internal gap between the restoration and the tooth provides the space required for the cement (Vojdani et al., 2013). The lower the discrepancy, the lower the probability of failure of the treatment plan apart from the environmental and health factors (Dahl et al., 2017; Kane et al., 2015; Park et al., 2017). Theoretically, the amount of internal space required for cementing is 20–40 μm. According to a study by Fransson et al., cement layer thicknesses in the range of 25–50 μm are rarely seen in the clinic (McLean, 1971). Mclean reported that gaps and internal spaces of less than 120 μm were clinically acceptable (McLean, 1971).

Some studies compared the internal fit of CAD/CAM and other fabrication methods of PFM crowns and reported controversial results, which could be related to their evaluation methods (Jung, 2017; Nesse et al., 2015). A variety of methods have been used, such as silicon replica (Jung, 2017; Nesse et al., 2015), digital techniques (Dahl et al., 2017; Kane et al., 2015), replica weighting (Kim et al., 2017), and sample sectioning after cementing in studies investigating the internal fit of PFM crowns for cobalt–chromium alloys (Kim et al., 2017). In recent years, triple scanning techniques have been used to evaluate the internal fit by measuring the difference between images through different scans (Dahl et al., 2017; Kane et al., 2015; Park et al., 2017).

This study was conducted to evaluate the internal fit of cobalt–chromium PFM crowns fabricated with lost wax and CAD/CAM techniques at the framework stage, after porcelain application, and after cementation using digital triple scanning, replica weighting, and observation with scanning electron microscopy. There were two null hypotheses: (1) internal fit of PFM crowns was the same in both fabrication methods (lost wax and CAD/CAM). (2) Internal fit of PFM crowns was the same in all fabrication steps (framework, porcelain, after cementation).

## 2 MATERIALS AND METHODS

### 2.1 Sample preparation

**Figure 1:**
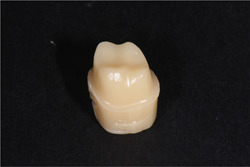
Sample prepared for study

In this in‐vitro experimental study, a maxillary first molar model of the typodont (Nissin, Dental Product) was prepared for a metal‐ceramic crown with the following specifications: 1.2 mm wide radial slopping shoulder finish line, 1.5 mm occlusal surface reduction from the functional cusp, and 1 mm from nonfunctional cusp, and 1.5 mm functional cusp bevel at 45° with 6° convergence. This typodont was scanned (Open Technologies‐Deluxe optical 3D scanner) and the 20 similar resin models (PMMA milling disk, Yamahachi Dental) were milled out of it (Figure 1). Some indentations were created using a bur (Tapered Fissure Bur 171L‐012) in the base of the resin models beyond the finish line as reference points. Impression making was performed concurrently with the polyvinylsiloxane Putty‐Wash method (X‐light Panasil‐Kettenbach), and the prepared gypsum dies (Vel‐Mix die stone. Gypsum. Type 4) were randomly divided into two groups: lost wax (conventional) and CAD/CAM.

### 2.2 Framework fabrication

In the lost wax group, a spacer (Renfert die: master) was applied to gypsum dies with a thickness of 30 μm (2 layers of 15 µm spacer), 1 mm away from the finish line. Then, a 0.5 mm wax‐up cutback coping was prepared (Renfert GMBH) with a lingual metal shoulder (1 mm height and 0.5 mm width) (Morrow & Rudd, 1986). Patterns were sprued with a 45° angle to the occlusal surface and placed close to the center of the ring. It was then invested with phosphate‐bonded investment (Z4) with 24 ml liquid to 100 gr of powder ratio and casted with cobalt–chromium alloy (Bego Wirobond 280), and the casting rods were cut off using tungsten carbide bur. Frameworks were blasted with 50‐μm glass beads (Perlablast®, Bego) with a pressure of 2 bar, in 10 cm distance and 90° angle for 2 s. The metal frames were polished with rubber polisher and brushes. In the CAD/CAM group, gypsum dies were scanned by a laboratory scanner (Open technologies optical 3D scanner) and the frameworks were designed using the Exocad‐Germany software according to the features mentioned in the previous group (30‐µm cement gap). The design was sent to the milling machine and the frameworks were fabricated from the hard sintered cobalt–chromium block (ARUM‐Korea) with Arum 5 × 200 milling machine (5 Axis, wet milling, with 1.5 mm diameter bur and burs were replaced after 15 units milling).

**Figure 2:**
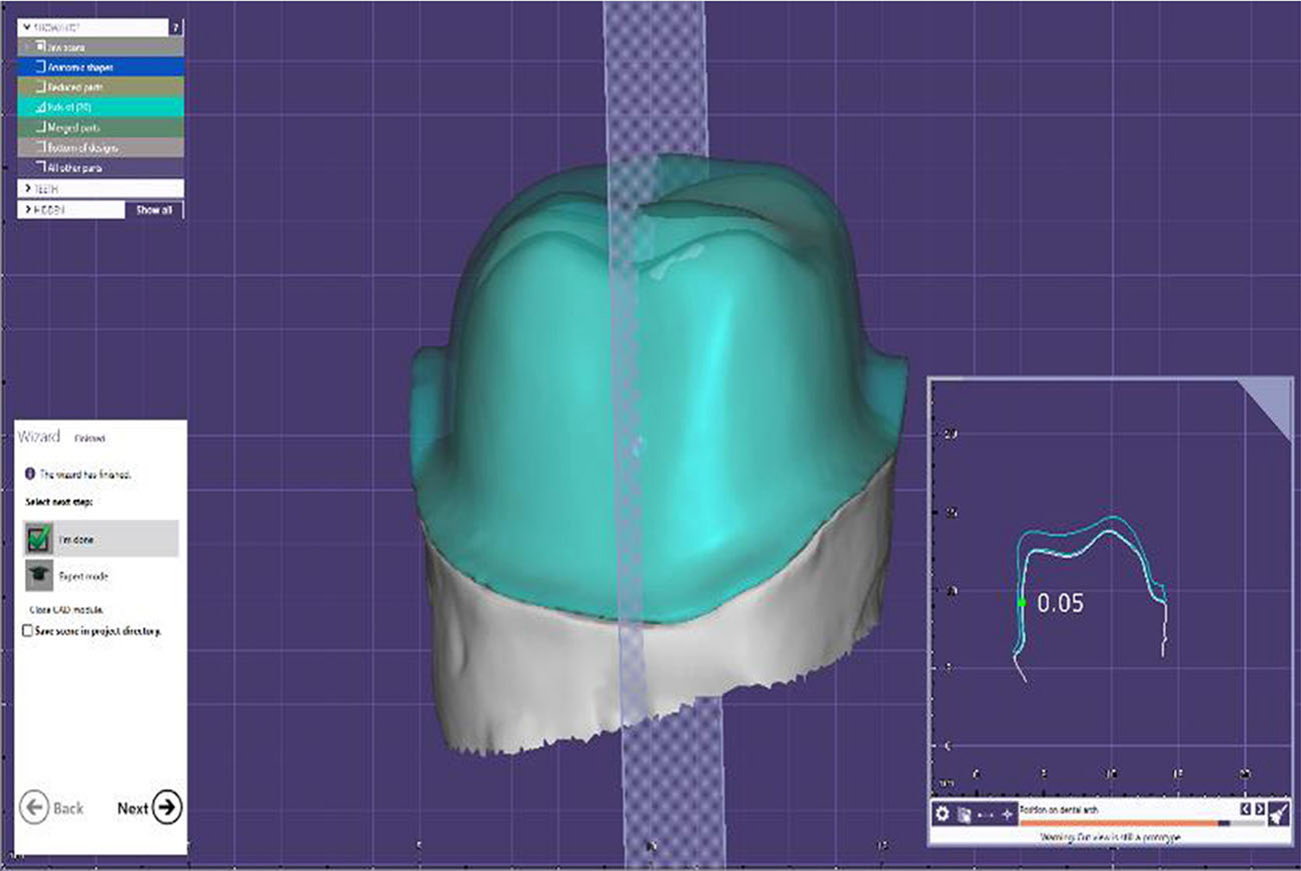
Triple scanning method for internal fit evaluation

**Figure 3:**
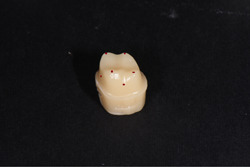
Measurement points in triple scanning method; tip of four occlusal surface cusps and midpoints of axial walls

**Figure 4:**
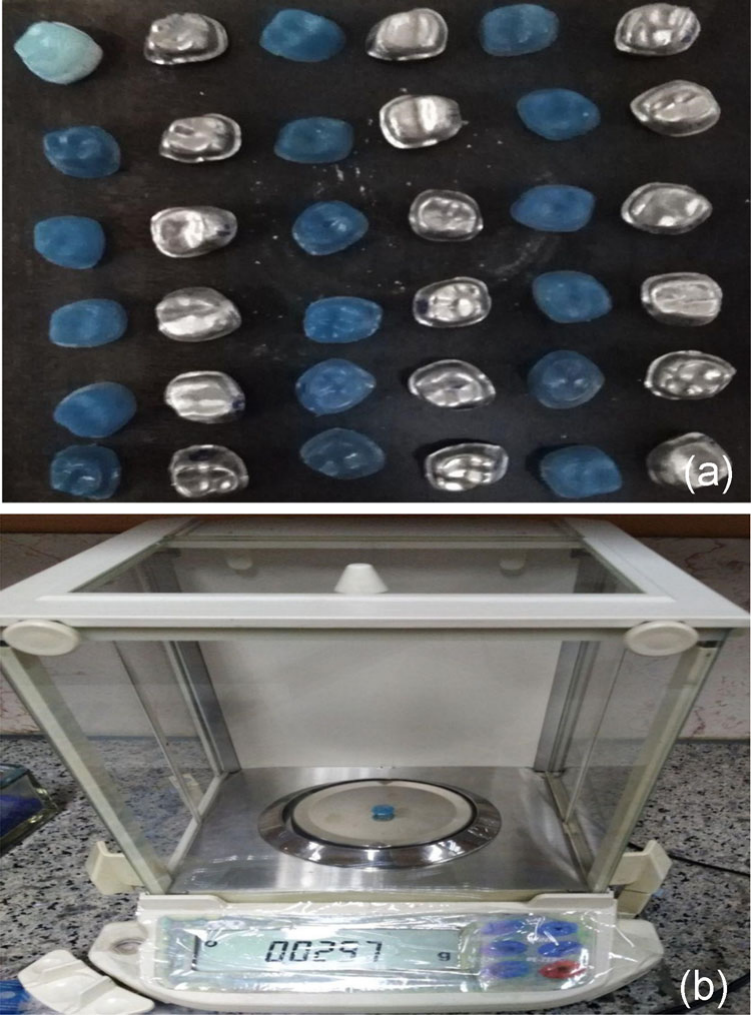
Replica weighting method: (a) prepared replica and (b) digital scale

The internal fit of the frameworks in both groups was evaluated using the following methods:

− Triple scanning method: A layer of powder (Renfert ScanSpray 1 × 200 ml 6.76) was sprayed on the outer surface of all resin dies and the inner and outer surfaces of all‐metal frameworks prepared in both groups and then these surfaces were scanned (Open technologies optical 3D scanner). Each framework was then mounted on its die, and the outer surface was scanned in full‐framework placement on the resin die. The three scans were superimposed through reference points on the die and framework using the Automatic Adjustment and Alignment System (Exocad) (Figure 2). After matching the images obtained from triple scanning, the internal fit was evaluated at eight points (at four occlusal surface cusps and midpoints of axial walls at buccal, palatal, mesial, and distal surfaces) (Figure 3).
− Replica weighting method: Using a light body of condensational silicon replica material (Coltene speedex), the metal frameworks were placed completely on the related resin dies with a vertical force of 10 N applied by a 1 kg weight. Immediately after the restorations were placed on the die, the replica excess was removed using dry clean cotton rolls. After setting time, replica materials were gently removed from the inner surface of the framework and weighed by a digital scale (AND GR 202) with an accuracy of 10^−4 ^g (Figure 4), and the internal fit of the two groups was compared using this method.

### 2.3 Porcelain application

Subsequently, the porcelain application was performed on the metal copings in both groups through layering technique as opaque (Kuraray Noritake), dentin (Kuraray Noritake), and enamel shades (Kuraray Noritake) in a furnace (KoushaFan Pars AT‐300) using a silicon index prepared by putty from a maxillary molar sample. The prepared crowns were evaluated again for the internal discrepancy using triple scanning and replica‐weighting methods as mentioned before.

### 2.4 Cementation

**Figure 5:**
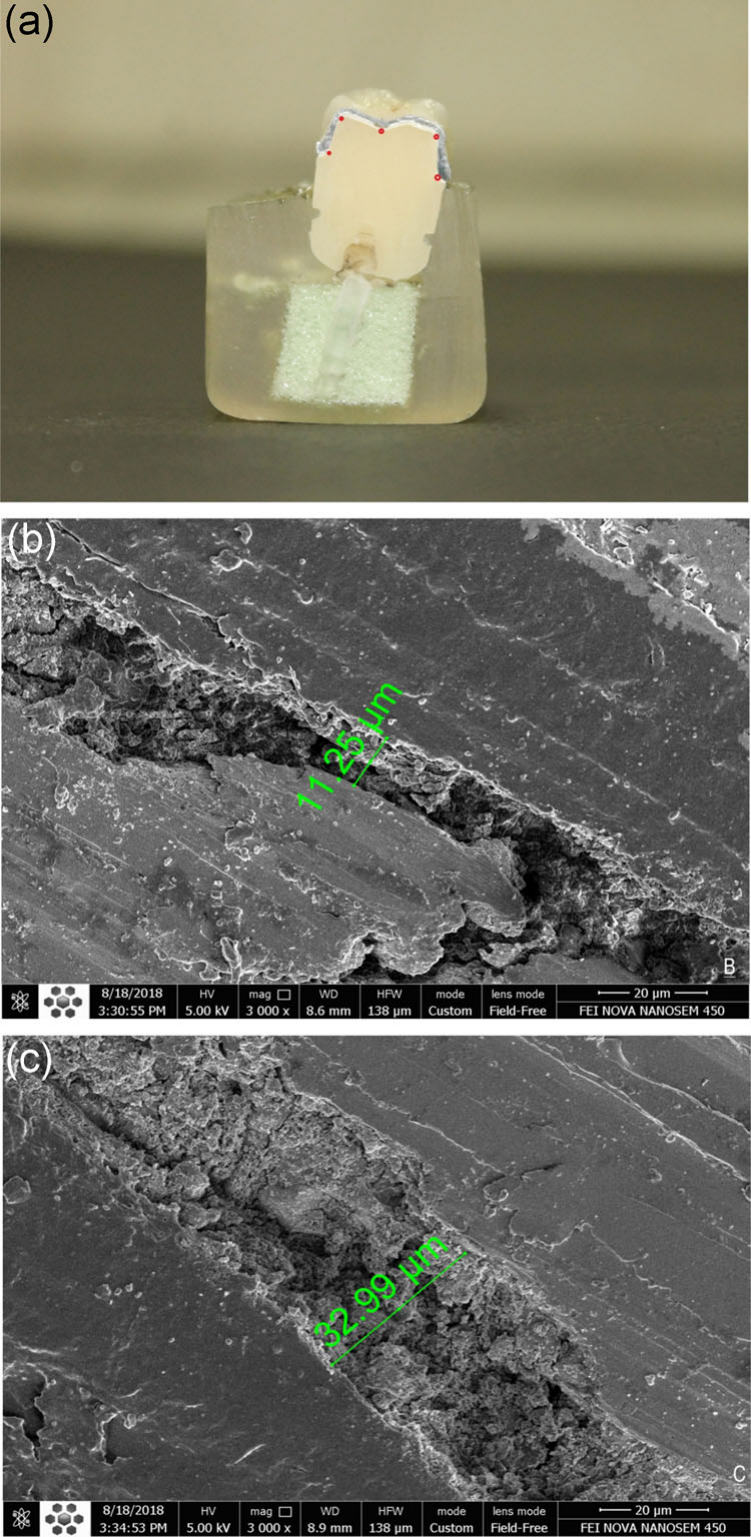
Scanning electron microscope analyzing: (a) a sectioned sample and measurement points, (b) internal space in occlusal surface, (c) internal space in axial surface

The final prepared crowns in both groups were cemented to the die using zinc phosphate cement (Hoffmann's Zinc Phosphat Cement) as the gold standard (Pameijer, 2012) and a force of 10 N was applied vertically using a 1‐kg weight until the cementing stage was complete. Then, internal discrepancy assessment was repeated using the triple scanning method. Finally, the samples were mounted vertically in polyester material and sectioned mesiodistally using a double‐blade diamond disc with a 0.3‐mm thickness at a speed of 2500 rpm (MECATOME T 201 A‐PRESI‐France). The internal fit assessment was performed by direct visualization using a scanning electron microscope (FEI Nova nanoSEM 450‐USA). Five points (two axial line‐angle points, two occlusal line‐angle points, and one occlusal midpoint) were measured using the QUANTAX micro‐XRF‐USA software (Figure 5).

### 2.5 Data analysis and statistic

Data were analyzed using SPSS version 22. Independent‐samples *t* test was applied to evaluate significant differences at each step (framework, porcelain, and cementation). One‐way analysis of variance (ANOVA) was used to evaluate discrepancies in different steps between the two groups (lost wax and CAD/CAM). Correlation analysis of different measurement methods was done using the Pearson correlation test. The significance level was set at 0.05.

## 3 RESULTS

The data obtained from the fit assessment in different stages, including framework, porcelain, and after cementation, were compared using triple scanning, replica weighting, and electron microscopy.

### 3.1 Framework

Independent *t* test showed no significant difference in the internal fit between the two groups using triple scanning and replica weighting (*p* > .05) (Table 1). The mean internal gap in the occlusal area was 53.08 μm in the CAD/CAM group and 69.27 μm in the lost wax group, but the independent *t* test showed no significant difference (*p* = .29). Moreover, the mean internal gap in the axial area was 35.00 μm in the CAD/CAM and 47.70 μm in the cast group, indicating no significant difference (*p* > .05).

**Table 1:** Mean internal gap of CAD/CAM and lost wax crowns in framework step

Evaluation method	Study groups	Mean internal gap	Standard deviation	*p*
Replica weighting	CAD/CAM	0.036 g	0.012	.10
	Lost wax	0.044 g	0.008	
Triple scanning	CAD/CAM	44.04 μm	12.99	.65
	Lost wax	58.48 μm	18.18	

Abbreviation: CAD/CAM, computer‐assisted design/computer‐aided milling.

### 3.2 Porcelain

According to the independent *t* test, the replica weighting method showed that CAD/CAM group had a better internal fit (*p* < .001), while the triple scanning method revealed no significant difference between the two groups (*p* > .05) (Table 2).

**Table 2:** Mean internal gap of CAD/CAM and Lost wax crowns in porcelain step

Evaluation method	Study groups	Mean internal gap	Standard deviation	*p*
Replica weighting	CAD/CAM	0.024 g	0.005	<.001
	Lost wax	0.039 g	0.006	
Triple scanning	CAD/CAM	58.27 μm	29.44	.44
	Lost wax	49.76 μm	16.91	

Abbreviation: CAD/CAM, computer‐assisted design/computer‐aided milling.

The mean internal gap in the occlusal area was 93.36 μm in the CAD/CAM group and 61.45 μm in the lost wax group, indicating no significant difference (*p* = .16). Moreover, the mean internal gap for the axial area was 23.19 μm in the CAD/CAM and 38.07 μm in the lost wax group. According to the statistical analysis, the mean internal gap was significantly lower in the axial area of the CAD/CAM group compared to the cast group (*p* < .05).

### 3.3 After cementation

According to the results of the independent *t* test, triple scanning and scanning electron microscopy showed no significant difference between the two groups (*p* > .05) (Table 3).

**Table 3:** Mean internal gap of CAD/CAM and lost wax crowns after cementation

Evaluation method	Study groups	Mean internal gap	Standard deviation	*p*
Triple scanning	CAD/CAM	42.75 μm	4.80	.28
	Lost wax	40.73 μm	3.11	
Scanning electron microscopes	CAD/CAM	83.88 μm	55.39	.67
	Lost wax	74.97 μm	28.32	

Abbreviation: CAD/CAM, computer‐assisted design/computer‐aided milling.

According to the triple scanning method, the mean internal gap in the occlusal area was 52.27 in the CAD/CAM group and 53.35 μm in the lost wax group, indicating no significant difference. Moreover, the mean internal gap in the axial region was 32.22 in the CAD/CAM group and 28.12 in the cast group, which showed no significant difference (*p* > .05).

According to the scanning electron microscopy method, the mean internal gap in the occlusal area was 110.13 μm in the CAD/CAM and 103.106 μm in the cast group, which were not statistically significant. Furthermore, the mean internal gap in the axial region was 44.51 μm in the CAD/CAM and 32.85 µm in the cast group, which were not statistically significant (*p* > .05).

### 3.4 Internal discrepancy changes in fabrication steps

The mean internal gap of all samples was calculated in each step and method. The results were as follows:

Triple scanning method: One‐way ANOVA showed no significant difference in the mean total internal gap between the porcelain stage (53.79 μm) and the framework stage (51.64 μm) (Sig > 0.05). However, the mean internal gap showed a significant decrease following cementation (41.69 μm) (Sig < 0.05).

The mean internal gap measured in the occlusal area was 61.60 μm for all specimens at the framework stage, 76.56 μm at the porcelain stage, and 52.84 μm after cementing. One‐way ANOVA showed no significant difference in the mean internal gap measured in the occlusal area between the porcelain stage and the framework stage (Sig > 0.05), while there was a significant decrease in the gap after being cemented in comparison with the porcelain stage (Sig < 0.05).

The mean internal gap measured in the axial area was 41.68 μm at the framework stage for all specimens, 31.02 μm at the porcelain stage, and 30.53 μm following cementation. One‐way ANOVA showed that the gap reduced significantly following porcelain application in the axial area (Sig < 0.05). However, the was no significant difference in the mean internal gap measured in the axial area between the porcelain application stage and after cementation (Sig > 0.05).

Replica weighting method: The mean replica weight was 0.040 g in the framework stage and 0.032 g in the porcelain stage. Paired samples *t* test results showed that the internal fit was significantly better at the porcelain stage compared with the framework stage (Sig < 0.05).

### 3.5 Correlation of different measurement methods

Correlation analysis of different measurement methods was performed using the Pearson correlation test and the results were as follows:

Replica/triple scan: There was no significant relationship between the values obtained from these two measurement methods in framework (correlation: 0.084, *p* = .733) and porcelain steps (correlation: −0.209, *p* = .392).

Scanning electron microscope/triple scanning: There was no significant relationship between the values obtained from these two methods in all samples after cementation (Correlation: −0.093, *p* = .704) (*p* > .05).

## 4 DISCUSSION

This study was conducted to evaluate the internal fit of cobalt–chromium PFM crowns fabricated by lost wax and CAD/CAM techniques in the metal framework, porcelain, and after cementation stages through digital triple scanning, replica weighting, and sectioning followed by visualization under a scanning electron microscope. The first null hypothesis was approved and the results showed that the internal fit of PFM crowns was similar in both fabrication methods (lost wax and CAD/CAM). The second null hypothesis was rejected and the internal fit of PFM crowns was not the same in different fabrication steps (framework, porcelain, after cementation).

Few studies have evaluated internal fit in comparison to the large number of studies that have investigated the marginal fit of fixed prostheses. The reason could be related to conventional evaluation methods. For marginal fit evaluation, nondestructive methods such as direct visualization under a microscope are routinely used, while the conventional method for internal fit evaluation is destructive requiring sectioning and visualization under a microscope. Although this method is accurate and precise, it is difficult, expensive, and two‐dimensional and requires sample destruction (Son et al., 2019). Some nondestructive methods were introduced in the last decades. The replica technique is popular. In this method, the silicon impression material is injected inside the prosthesis and removed after setting, sectioned, and observed under a microscope. This technique is relatively simple, fast, and inexpensive; however, the probability of deformation and tearing of impression material could make errors. Moreover, it is a two‐dimensional analysis (Son et al., 2019). Another nondestructive method is micro‐computer tomography, which provides a three‐dimensional image for internal fit evaluation. This technique is precise but has disadvantages such as the risk of radiation and artifact production in metallic restorations (Son et al., 2019).

In the present study, one of the evaluation methods was replica weighting, which is a technique for comparison between samples. To the best of our knowledge, there is no report of a certain weight for clinically acceptable internal fit. The results showed no difference between CAD/CAM and lost‐wax groups in the framework step, which is in contrast to a study by Kim et al that reported better fit in the lost‐wax group. This difference could be related to differences in the brands of Co–Cr alloy, CAD/CAM system, silicon material, and the analytical balance used (Kim et al., 2017). After porcelain application, the internal fit was significantly better in the CAD/CAM group and all samples in both groups became fitter in comparison to the framework step. This result was not expected and it could be related to technique sensitive manipulation of silicon in terms of amount of catalyst, speed of mixing, and removing the residuals, however all steps were done by one clinician. On the other hand, this result is according to triple scanning findings, which revealed less axial space and more occlusal gap in the porcelain step, which might result in less internal room for silicon material.

Triple scanning is a nondestructive method with no radiation. We scanned the internal and external surface of restoration, abutment, and the crown mounted on the abutment. By superimposition of these scans, the internal fit could be measured three‐dimensionally. The triple‐scan method is safe and minimizes manual errors that probably occur in other measurement methods such as the replica method. Nonetheless, this method requires costly scanner equipment and extensive scanning processes (Dahl et al., 2018a). Based on the triple scan results, there was no significant difference in the internal fit between CAD/CAM and lost‐wax techniques in different fabrication steps (framework, porcelain, and after cementation). The internal gap after cementation was 42.75 and 40.73 μm in CAD/CAM and lost wax groups, respectively, which were both clinically acceptable according to the Mclean study (less than 120 μm) (McLean, 1971). In 2017, Dahl et al. also used triple scanning to compare the internal fit of single crowns and found that the overall internal fit was similar in CAD/CAM and lost‐wax groups, which was consistent with our findings (Dahl et al., 2018b). Gürel et al. reported that the mean internal gap was lower in Co–Cr traditional casting compared to the milling group but the difference was not statistically significant, which is in line with the results of the present study (Gurel et al., 2019).

The triple scanning method is an objective tool to observe internal fit changes in fabrication steps. Based on the results, internal fit increased in the porcelain stage compared to the framework stage due to the porcelain firing process. However, this increase was not significant. On the other hand, the internal fit was significantly lower after cementation compared to the porcelain stage, which could be due to the cementing procedure.

The last evaluation method was sectioning and visualization under a scanning electron microscope. According to Nawafleh et al. (2013), this technique is superior in terms of accuracy, imaging quality, and relative accuracy of the data due to the high distinction between different materials in the resulting images; however, it results in sample destruction and, therefore, this technique was only used in the final step after cementation in the present study. Scanning electronic microscopy showed that the mean internal gap was not significantly different between the CAD/CAM and lost wax restorations, which is not consistent with studies conducted by Dahl et al. reported a markedly higher internal fit in the conventional lost‐wax group (Dahl et al., 2017, 2018b), which could be related to different measurement methods and the limited number of samples. However, this finding is not consistent with a systematic review conducted by Per Svanborg et al.; they found a higher internal gap in the lost‐wax group in comparison with the CAD/CAM group (Svanborg & Hjalmarsson, 2020).

The mean internal fit measured by electron microscopy following cementation was 83.88 μm in the CAD/CAM group and 74.97 μm in the casting group. This amount of internal fit is acceptable according to a study by Mclean (McLean, 1971) (<120 μm).

Gap size in occlusal and axial surfaces was measured separately by triple scanning and electron microscopy. No significant difference was found in the occlusal and axial gap between the two groups, and the occlusal gap space was higher than the axial gap space in all fabrication steps.

In addition to evaluating the internal fit of metal‐ceramic restoration and comparing the fabrication methods, the present study compared the proximity of data obtained from different measurement methods. There was no correlation between replica weighting and triple scanning, which is inconsistent with a study by Svanborg et al.; they claimed that the impression replica technique might be as precise as the triple‐scan method for internal fit measurement of tooth‐supported restorations (Svanborg et al., 2019).

The triple scanning method had no significant correlation with the electron microscopy measurement method. Given the superiority of the scanning electron microscope for evaluating the internal fit (Nawafleh et al., 2013), the triple scanning method may not be trusted in PFM restorations due to scan inaccuracy in high polish metallic surfaces and nonuniform application of the scan powder (Kuhn et al., 2015). As a solution, Dahl et al. proposed the dual‐scan (digitized version of the impression replica technique) method for measuring internal adaption. In their opinion, dual‐scan was more rapid and simplified than triple scanning for evaluating internal discrepancy (Dahl et al., 2018b).

For simulating the best probable accuracy, most of the investigators use an in‐vitro study design, but these studies cannot simulate clinical situations because of challenges, such as salivary flow, oral moisture, and thermal and loading cycling. On the other hand, we did not use human teeth in this study. Examining just one CAD/CAM system, one type of finishing line, and laboratory skills added to the limitations of this study. Due to these points, caution should be exercised in generalizing the results of this study to the clinic.

To compare the accuracy and success of different fabrication techniques and fit evaluation methods, studies are currently at the beginning of the route. Considering the limited clinical trials in this area and the need for evaluation of long‐term follow‐up results to prove the success of a clinical approach, the advances of new digital systems, the importance of proper performance, and awareness of these systems' errors, it seems that more studies are necessary to further investigate laboratory materials, design systems, and comparative assessment methods.

## 5 CONCLUSION

According to the results of scanning electron microscopy as the superior evaluation method, the internal fit of cobalt–chrome PFM crown of both CAD/CAM and lost wax groups was within the acceptable clinical range and there was no significant difference between the two groups. Triple scanning methods revealed no difference between the two groups in all steps. Also, it showed no difference in the internal fit of framework and porcelain steps but a better fit after cementation. Replica weighting recorded no difference between the two groups in the framework step and better fit for CAD/CAM group in the porcelain step. According to replica weighting, the internal fit in the porcelain step was higher than the framework. The results of triple scanning and replica weighting had no significant correlation with the scanning electron microscopy, and therefore the results of these two methods are not reliable.

## CONFLICT OF INTERESTS

The authors declare that there are no conflict of interests.

## AUTHOR CONTRIBUTIONS

*Conceptualization, data curation, project administration, and article editing*: Dr Elham Ahmadi and Dr Masoumeh Hassani Tabatabaei. *Investigation, methodology, formal analysis, writing–original draft*: Dr Sina Mohammadi Sadr. *Writing–original draft, and editing*: Saba Mohammadi. *Conceptualization, supervision, data curation, funding acquisition, investigation, project administration, methodology, formal analysis, and article editing*: Dr Faezeh Atri.

## Data Availability

The data that support the findings of this study are available on request from the corresponding author.
